# Towards deep learning based smart farming for intelligent weeds management in crops

**DOI:** 10.3389/fpls.2023.1211235

**Published:** 2023-07-28

**Authors:** Muhammad Ali Saqib, Muhammad Aqib, Muhammad Naveed Tahir, Yaser Hafeez

**Affiliations:** ^1^ University Institute of Information Technology (UIIT), Pir Mehr Ali Shah (PMAS)-Arid Agriculture University Rawalpindi, Rawalpindi, Punjab, Pakistan; ^2^ National Center of Industrial Biotechnology, Pir Mehr Ali Shah (PMAS)-Arid Agriculture University Rawalpindi, Rawalpindi, Punjab, Pakistan; ^3^ Department of Agronomy, Pir Mehr Ali Shah (PMAS)-Arid Agriculture University Rawalpindi, Rawalpindi, Punjab, Pakistan; ^4^ Pilot Project for Data Driven Smart Decision Platform for Increased Agriculture Productivity, Pir Mehr Ali Shah (PMAS)-Arid Agriculture University Rawalpindi, Rawalpindi, Punjab, Pakistan

**Keywords:** artificial intelligence, digital agriculture, object detection, weed management, YOLO

## Abstract

**Introduction:**

Deep learning (DL) is a core constituent for building an object detection system and provides a variety of algorithms to be used in a variety of applications. In agriculture, weed management is one of the major concerns, weed detection systems could be of great help to improve production. In this work, we have proposed a DL-based weed detection model that can efficiently be used for effective weed management in crops.

**Methods:**

Our proposed model uses Convolutional Neural Network based object detection system You Only Look Once (YOLO) for training and prediction. The collected dataset contains RGB images of four different weed species named Grass, Creeping Thistle, Bindweed, and California poppy. This dataset is manipulated by applying LAB (Lightness A and B) and HSV (Hue, Saturation, Value) image transformation techniques and then trained on four YOLO models (v3, v3-tiny, v4, v4-tiny).

**Results and discussion:**

The effects of image transformation are analyzed, and it is deduced that the model performance is not much affected by this transformation. Inferencing results obtained by making a comparison of correctly predicted weeds are quite promising, among all models implemented in this work, the YOLOv4 model has achieved the highest accuracy. It has correctly predicted 98.88% weeds with an average loss of 1.8 and 73.1% mean average precision value.

**Future work:**

In the future, we plan to integrate this model in a variable rate sprayer for precise weed management in real time.

## 1 Introduction

As the world’s population is growing drastically, food deprivation is increasing worldwide (Gilland, 2002). To cope with the deficiency in food quantity, we need to increase crop yield. Modern agricultural practices like precision agriculture, smart farming, food technology, plant breeding, etc. are using smart technology (Liang and Delahaye, 2019; García et al., 2021; Hati and Singh, 2021; Mavani et al., 2021; van Dijk et al., 2021; Zhang et al., 2021) to intensify crop production. In smart agriculture, artificially intelligent systems are incorporated for making smart decisions, to increase crop yield (Hague et al., 2006; Franco et al., 2017). The major components that affect the crop yield are diseases of plants, irrigation system, application of agrochemicals, pest infestation, and weeds, etc. (Oerke, 2006; Mitra, 2021; Reginaldo et al., 2021; Jastrzebska et al., 2022). Only weeds have caused an economic loss of about 11 billion USD in 18 states of India between the years 2003 and 2014 (Gharde et al., 2018). Automated weed control and management systems can reduce the yield loss up to 50% and above (Dass et al., 2017).

Automated detection and identification of weeds is the first phase in the development of weed reduction system (Fernández-Quintanilla et al., 2018; Munz and Reiser, 2020). DL algorithms are better for image based classification and object identification tasks. They are mainly built on neural networks and are well known for pattern recognition in image (Szegedy et al., 2015; Guo et al., 2016; Shrestha and Mahmood, 2019). These deep neural networks have many hidden layers and each hidden layer performs some operation on input data, which leads to the identification of the object. DL based algorithms are widely used in all kind of research problems in the field of medical diagnosis (Bakator and Radosav, 2018; Yoo et al., 2020), smart traffic management (Aqib et al., 2018; Aqib et al., 2019a; Aqib et al., 2019b), and specifically in smart crop management practices (Balducci et al., 2018; Mohamed et al., 2021). They are producing considerable results in pest detection (Khalid et al., 2023), weeds detection (Khan et al., 2022) etc. using object detection in the agricultural field.

In this paper, we present a DL-based object detection model for weed detection in the agricultural field. For this purpose, a deep convolutional neural network (CNN) based YOLO object detection system is employed. To perform this experiment, the dataset was collected in the agricultural field during different time intervals and light conditions. A dataset of four weed species was collected by using image sensors. This dataset was pre-processed before using it for the training of different models of the YOLO object detection system. The models were trained and evaluated using different model configuration settings. An unseen data set was prepared to validate all the trained models using performance matrices. To the best of the author’s knowledge, we are the first to develop a detection system for the detection of the different weed species in a wheat field in the Pothohar region, Pakistan.

This paper’s contributions in the field of weeds management using DL includes the following:

1. Collection and preparation of real weeds dataset, collected from fields in Pothohar, Pakistan.
2. After training YOLO models with different configurations, we have provided the best weed detection model with the best configuration settings.
3. A study of the application of image transformation techniques on model performance.
4. An analysis of the effects of data augmentation techniques on the prediction of objects.

The rest of the study is organized as follows: the review of research articles related to this domain is presented in Section 2. Then Section 3 discusses the process workflow, proposed methodology, and the input dataset, its preprocessing, and other related details. Detailed discussion on experimental Setup and prediction results is given in Sections 4 and 5, and finally we have concluded the study in Section 6.

## 2 Related work

Classification and detection of objects in agricultural fields for recognition of weeds has been a hot area of research. In this section, we have discussed some of the studies in which the detection and classification of weeds are performed using AI-based techniques like DL, computer vision, robotics etc.

In (Zhuang et al., 2022), a study is organized for the detection of broadleaf weed seedlings in wheat fields. They have concluded that FR-CNN, YOLOv3, VFNet, TridentNet, and CenterNet are not suitable for detection as their recall stays equal to or less than 58%. Whereas classification using AlexNet and VGGNet have produced above 95% F1-scores. In this study, the authors have used a very small image resolution of 200 × 200 px dataset for training the models.

A system was developed in (Potena et al., 2017) study to classify weeds using multi-spectral camera. They used BoniRob robot in the process of data collection (Chebrolu et al., 2017). This data set contained data collected from multiple sensors, including a 4-channel multispectral camera, RGB-D IR sensor, GPS, terrestrial laser scanner, and Kinect sensor. The data set consisted of data from the emergence stage to the stage where a robot can damage the crop. They used two models of CNN: a lightweight CNN was used for binary image segmentation, and a deeper CNN for classification purposes. They also proposed a clustering algorithm for making subsets of images that more closely resembled each other.

In (Madsen et al., 2020), the classification of weed was done on a data set consisting of 7590 images of 47 plant species. ResNet-50-v1 algorithm was used for classification and achieved an accuracy of 77.06% and recall of 96.79%. The data set was collected in a well-illuminated environment rather than collecting it in variable light conditions. In (Gao et al., 2020) study, they developed a detection model based on deep CNN known as YOLO architecture. They trained this model on 452 field images and tested with 100 images from a total of 2271 synthetic images collected of C. sepium and sugar beet. They compared YOLOv3 and YOLOv3-tiny and achieved a mean precision of 76.1% and 82.9% respectively, with an inference time of 6.48ms.

A study was done on the detection of weeds in perennial ryegrass with DL (Yu et al., 2019). They applied several DL models which include VGGNet, GoogleNet, AlexNet, and DetectNet. They had collected 33086 images of dandelion, ground-ivy, spotted spurge, andperennial ryegrass. The data set had 15486 negative and 17600 positive images (containing weeds). They had trained the models individually for multi- and single-weed species. The analysis showed that VGGNet had the highest F1-score and recall score of 0.928 and 0.99 respectively. DetectNet had the highest F1-score value which is above 0.98. Overall, in their experiment VGGNet and DetectNet performed better.

Classification of weeds was done using Naïve Bayes in (Giselsson et al., 2017) study, in which they had created a dataset consisting of approximately 960 plants belonging to 12 species. Data was captured in different growth stages. Images were gathered over the course of 20 days with an interval of 2-3 days. Images were captured from 110-115 cm above the ground. A total of 407 images were captured with a resolution of 5184 × 3456 px. They concluded that this classifier can only be applied to images with similar features.

In (Le et al., 2020), they performed two image enhancement techniques, local binary pattern and plant leaf contour mask on weeds data set to increase performance of classifier. Two data sets were used in that experiment, Bccr-segnet and Can-rad dataset. They performed classification using Support Vector Machine and achieved an accuracy of 98.63% with 4 classes. In (Hoang Trong et al., 2020), a classification approach using the late fusion of multi-model Deep Neural Networks (DNNs) was developed. They experimented with the Plant Seedlings and weeds data sets with 5 DNN models named as NASNet, Resnet, Inception–Resnet, Mobilenet, and VGG. Two data sets were used having 208477 images. The analysis showed that the methods achieved the best accuracy of 97.31% on the plant seedlings dataset and 98.77% accuracy on the CNU Weeds dataset.

In (dos Santos Ferreira et al., 2017), they carried out a study in Campo Grande, Mato Grosso do Sul, Brazil to perform weed detection in soybean crop using CNN. A data set was created using a drone, consisting of 15000 images but after preprocessing 400 images were selected. They carried out this study in five phases. Firstly, collection of data, then classifying the images using the superpixel algorithm. Thirdly, feature extraction based on color, shape, and texture. The fourth stage consisted of training of CNN classifier. The last stage consisted of returning the visual segmentation and classification results. The images were taken from an RGB camera with a size of 4000 × 3000 px and an altitude of 4 m. They applied ConvNets, Support Vector Machines, AdaBoost, and Random Forests, and ConvNet achieved the best accuracy of 98%.

In (Jiang et al., 2020), they proposed a combination of graph convolutional network and VGG16, ResNet-101, and AlexNet for the classification of weeds. Four data sets of corn, lettuce, radish, and mixed were used. The mixed dataset was constructed by combining corn, lettuce, and radish datasets. The proposed GCN approach was favorable for multi-class crops and weeds recognition with limited labeled data. They compared GCN-ResNet-101, GCN-AlexNet, GCN-VGG16, and GCN-ResNet-101 approaches and achieved accuracies of 97.80%, 99.37%, 98.93% and 96.51% respectively.

A prototype of All Terrain Vehicle (ATV) was developed for precise spraying in (Olsen et al., 2019). A data set was prepared consisting of 17,509 labeled images of eight different species of weeds as chine apple, Lantana, Parkinsonia, Parthenium, Prickly acacia, Rubber Vine, Siam weed and Snake weed. This data set was prepared in Australian region and is publicly available on (Olsen et al., 2019). About a thousand images of each species were captured with a high-resolution camera and GPS to track progress. Two DL CNN models were used, inceprion-v3 and ResNET-50, to set a baseline performance on the dataset. Both models were evaluated using performance matrices like inference time, pre-processing time, total inference time, and frame rate. They achieved an accuracy of 95.1% and 95.7% respectively in classification. The results achieved were good, but the prototype ATV was not evaluated in real fields.

In (Bosilj et al., 2020) study, they examined the role of transfer learning in different crops and weed detection, on three data sets, Sugar beets, Carrots, and Onions. They found that the training time was reduced up to 80% even if the data was not perfectly labeled, and the classification result had a 2% error ratio.

Weed detection was performed using transfer learning in (Espejo-Garcia et al., 2020). They merged DL and ML models for weed identification. DL models include Xception, Inception-Resnet, VGNets, Mobilenet, and Densenet. And ML models were Support Vector Machines, XGBoost, and Logistic Regression. They collected 1268 images of two crops and two weed species. They found that DenseNet and SVM achieved the highest F1-score measure of 99.29%.

An FCN-8s model was trained for semantic segmentation using synthetic hierarchical images (Skovsen et al., 2019). A data set was collected, having 8000 synthetic images of ryegrass, red clover, white clover, soil, and weeds. The mean intersection over union value was calculated between ground truth images and predicted images, and scored 55.0%. The IoU values of weed and soil classes were below 40%.

Semantic segmentation of weeds was performed using SegNet (convolution neural network for semantic segmentation) in (Lameski et al., 2017). To separate the plant pixels from ground pixels, Excess Green minus Excess Red (ExG-ExR) index was used. It improved the detection of plants. They collected a carrot weed data set which was comprised of 39 images of size 10 MP from approximately 1m height. Such large-size images were converted into smaller images using a sliding window approach. The data set was imbalanced as images of carrots were more than weeds.

In the prior work done on the development of weed detection systems, different ML and DL models are trained to classify, detect, and semantically segment weeds using various data sets. A variety of sensors and computing systems were employed to collect diverse data sets and train the models. It can be seen in the related work that there is a lack of implementation of image processing and image transformation techniques applied to the dataset to get better results. In this study, we have developed a weed detection model to detect four weed species grown in wheat fields. For this purpose, we have used the latest state-of-the-art object detection models, image processing, and image transformation techniques to achieve better results as compared to other studies presented in this section.

## 3 Materials and methods

**Figure 1:**
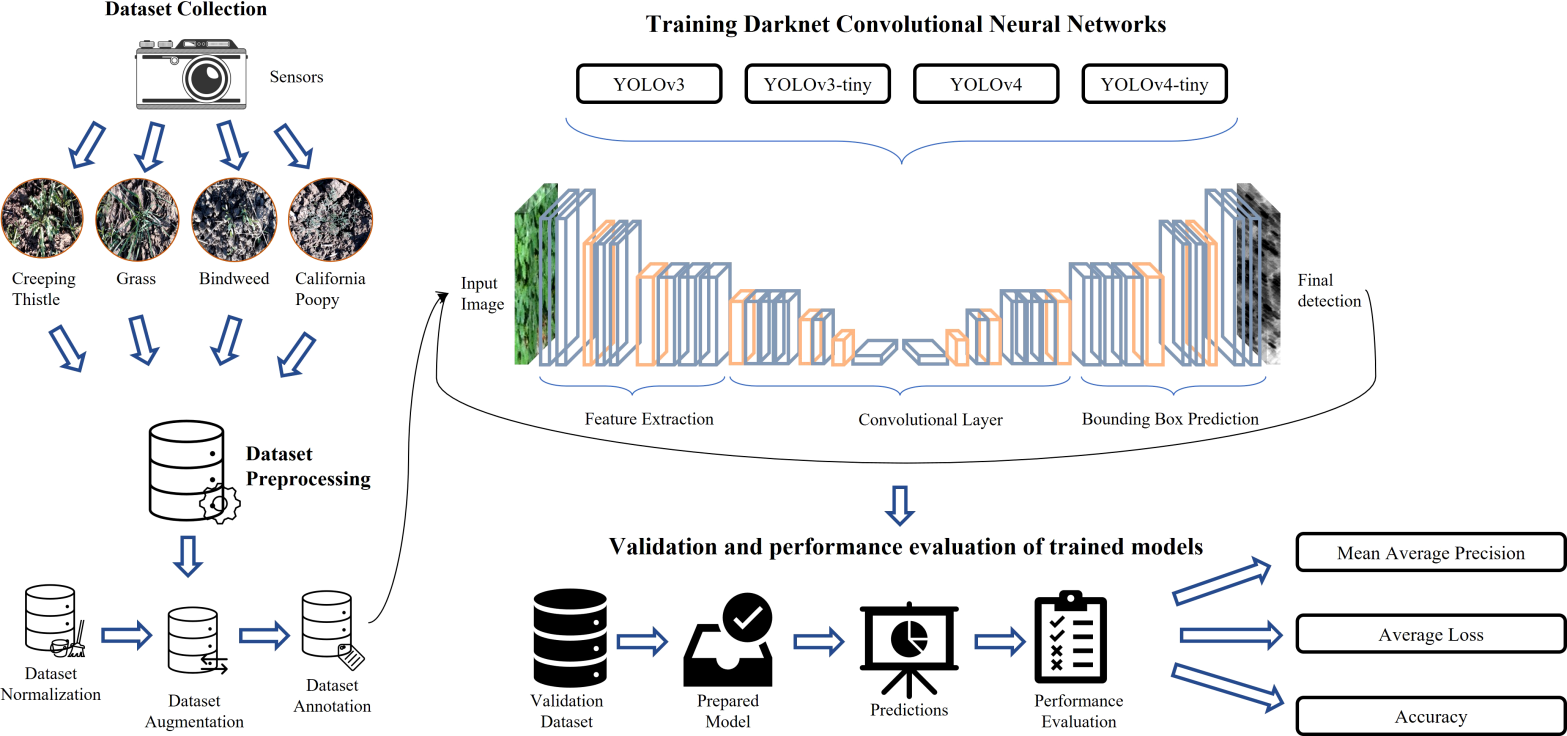
Process workflow of our weed detection system.

In this section, the steps involved in the whole experimental setup are elaborated. The experiment is designed to evaluate the capability of YOLO models and the effects of image processing for the detection of weeds in the real field. The workflow of our proposed methodology is illustrated in **Figure 1**. This figure highlights different phases of our proposed research methodology, which includes data collection, data pre-processing, model training, validation, and evaluation of prediction results. A brief overview of these phases is given in the following paragraphs.

In the data collection phase, data is collected from agricultural fields using cameras in different time stamps, environmental conditions, and lightning conditions. This data is prepared to be used in further processes like training, testing, and validation of models, etc. This phase is divided into three sub-phases, normalization, augmentation, and annotation. In normalization, data is regularized and checked for removal of irrelevant, uniform, and duplicated images, etc. In the augmentation phase, the shape and size of data are altered to produce better results in further processing. In annotation, a label is assigned to each entity in data that serves as a piece of initial training information for models.

Model training process is carried out by applying the following YOLO deep neural network architectures.

1. YOLOv3
2. YOLOv4
3. YOLOv3-tiny
4. YOLOv4-tiny

These models are trained on the pre-processed data and hyper parameter tuning was performed on each model to get the best configuration. The trained models are validated on unseen data and the predicted outputs of the model are evaluated by using different performance matrices. They include precision, recall, mean average precision, and average loss. These matrices can help in analyzing results produced by the model on the validation set.

### 3.1 Dataset

**Figure 2:**
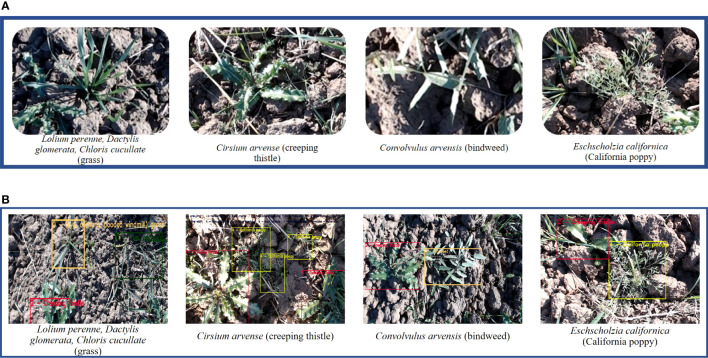
Illustration of **(A)** classes and **(B)** annotations.

The process of data collection is done in the winter season in the Potohar region, Pakistan. The data set collected consists of RGB images of different weed species grown in *Triticum* (wheat) field. These weeds are divided into four major classes which include *Lolium perenne*, *Dactylis glomerata*, *Chloris cucullata* (grass), *Cirsium arvense* (creeping thistle), *Convolvulus arvensis* (bindweed) and *Eschscholzia californica* (california poppy). The collected data set contains 1065 images collected using a Logitech HD 920c webcam pro camera with a resolution of 1 MP and dimensions of 1280 × 720. The data set is available at GitHub https://github.com/Aqib-Soft-Solutions/Wheat-Crop-Weeds-Dataset.git and a sample of it is illustrated in **Figure 2**.

**Figure 3:**
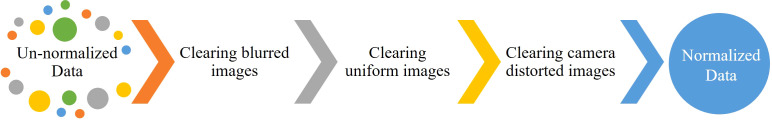
The process of data normalization.

After dataset collection, to make it ready for training, it needed to be normalized, augmented, and annotated first. To get better results on this data, we have performed certain cycles of training and changing data accordingly. The sub-processes of data pre-processing are described below. After analyzing data image by image, it was identified that it contains some issues that are needed to be resolved first. These issues are made by images that are blurred, uniform, camera distorted, etc. We have cleared such images that can potentially restrain our training process. The process of normalization is divided into sub-processes that are illustrated in **Figure 3**.

**Figure 4:**
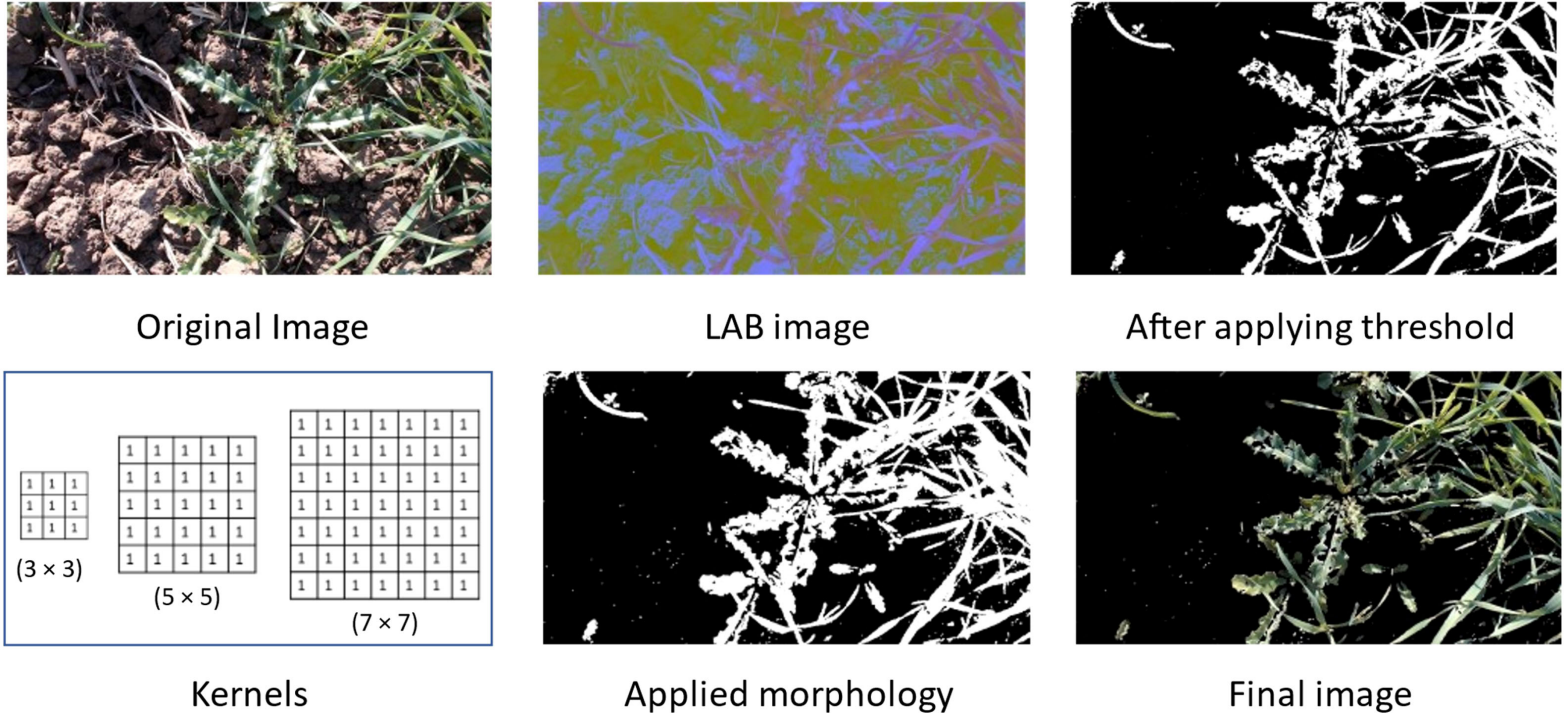
Applied masking using LAB color scheme.

**Figure 5:**
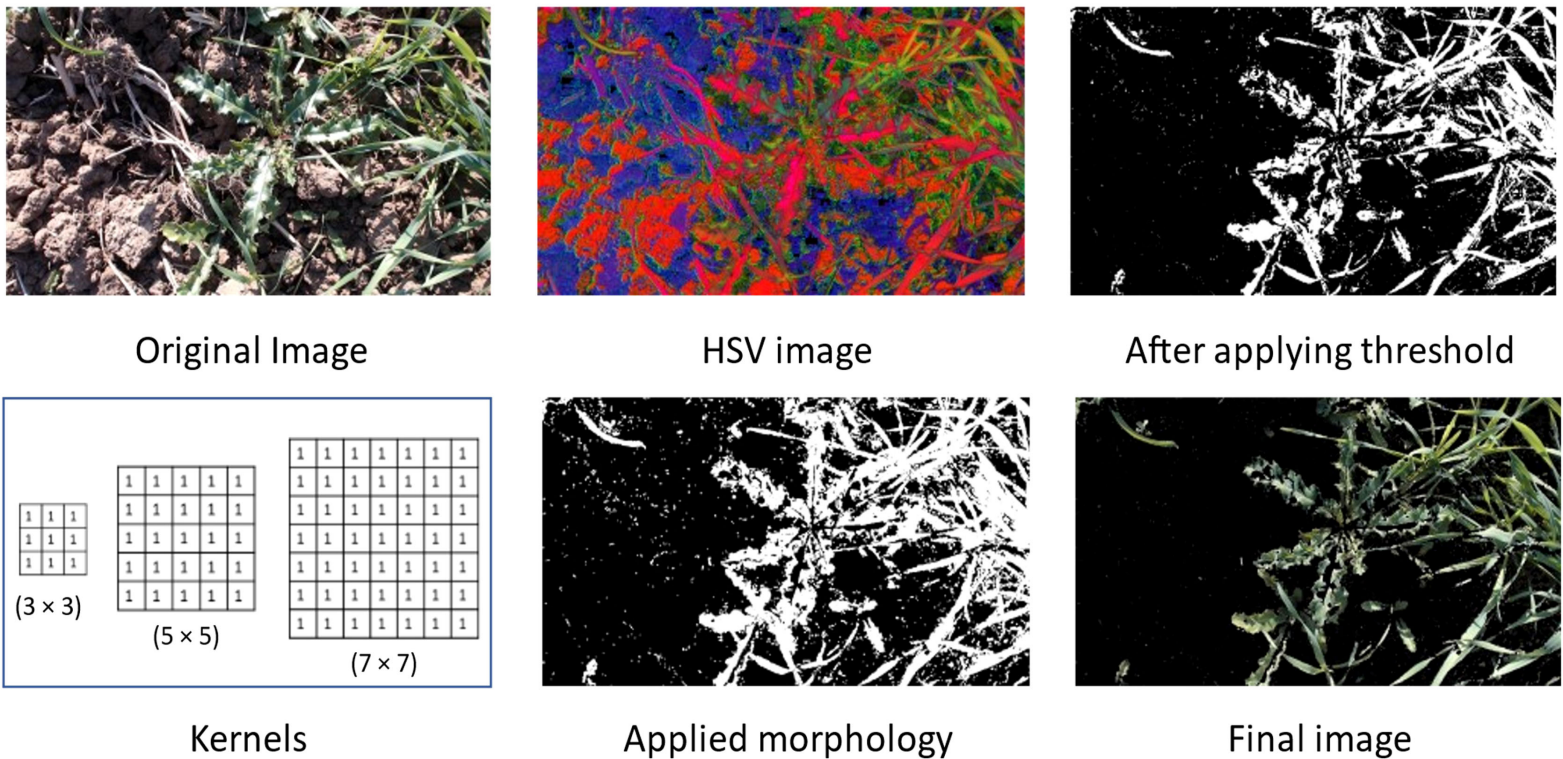
Applied masking using HSV color scheme.

We have applied different masking techniques to extract plants from the image. Image is converted from RGB to LAB and HSV color schemes, shown in **Figures 4**, **5** respectively. After converting in LAB, only the ‘b’ channel is preserved. Otsu’s binarization and binary invert thresholding are applied, in which the threshold is set to 105 and the max value to 255. Whereas, after converting the image in HSV, pixels in a range are extracted, where low and high values are set to (30, 25, 0) and (80, 255, 255) respectively.

After applying thresholding, the mask obtained has some missing portions of the main plant (object). To fill out these missing parts we have applied morphology on the mask and applied bitwise AND operation on that mask and original image to get the final resultant image.

The final image obtained after using the LAB color scheme has less noise than the image obtained after applying the HSV color scheme.

In the process of data augmentation, we performed augmentation on partial images of the dataset. Firstly, images are rotated at three different angles, 45, 90, and 180 degrees. Secondly, data is also altered with a saturation value of 1.5, exposure value of 1.5, and hue value of 0.1 during training.

In data annotation, a rectangular box is drawn around each object in the image and a label is assigned to it. To do this, we have used the YOLO mark. It produces a text file that contains information about each annotation in the image.

## 4 Results and evaluation

This experiment is organized to evaluate: (i) the performance of YOLO object detection systems to detect weeds; (ii) the results under different configurations of YOLO models; (iii) the effects of data augmentation on inferencing; (iv) effect of image masking on model performance. The whole process is carried out in multiple different scenarios as described below.

### 4.1 Performance matrices

In this section, we have described the evaluation matrices used to evaluate the results of YOLOv3, v3-tiny, v4, and v4-tiny models. The parameters used to evaluate results are described as follows,

#### 4.1.1 Precision

Precision is calculated for a particular class by dividing true positives by all positive predictions. We have used equation (1) to calculate the accuracy of trained model.


(1) $$Precision=\frac{True\;positives}{True\;positives\;+\;False\;positives}$$

#### 4.1.2 Recall

Recall of a class is calculated by dividing true positives and the sum of true positives and false negatives. We have used equation (2) to calculate the accuracy of trained model.


(2) $$Recall=\frac{True\;positives}{True\;positives\;+\;False\;negatives}$$

#### 4.1.3 F1-score

The F1-score is a measure of a model’s accuracy in classification tasks, especially when dealing with unbalanced data sets. It combines precision and recall into a single measure to provide a balanced assessment of model performance. We have used equation (3) to calculate the F1-score of the trained model.


(3) $$F1–score=\frac{2\cdot \left(Precision\cdot Recall\right)}{\mathrm{Pr}ecision+\mathrm{Recall}}$$

#### 4.1.4 Mean average precision

The mean average precision (mAP) is calculated by taking the mean of the average precision of every class. The average precision (AP) is a measure of the area under the precision-recall curve, calculated by using the formula in equation (4).


(4) $$AP\;=\;\int_{0}^{1}p\left(r\right)dx$$

Where *p*(*r*) is precision as a function of *r*. AP calculates average of *p*(*r*) over the interval of 0 ≤ *r* ≥ 1 (Zhu, 2004). To calculate the mAP, we have used the formula in equation (5).


(5) $$mAP\;=\;\frac{1}{n}\sum_{k=1}^{n}AP\left(k\right)$$

Where *n* is the total number of classes.

#### 4.1.5 Average loss

The average loss (AL) function of YOLO is the sum of classification loss, localization loss, and confidence loss which are calculated using equation (6) of Residual Sum of Squares (RSS) (Archdeacon, 1994). It is the deviation of predicted values from the actual ground truth values.


(6) $$RSS=\sum_{i=1}^{n}{\left(y_{i}-f\left(x_{i}\right)\right)}^{2}$$

Where, *RSS* = *Residual Sum of Squares*, *y_i_* = *i^th^* value of the variable to be predicted, *f* (*x_i_*) is the predicted value of *y_i_* and *n* is upper limit of summation.

### 4.2 YOLOv3 implementation

YOLOv3 is a well-known object detection system. We have evaluated its performance in the detection of weeds in different setups. Data is divided into three sets, train, test, and validation with a ratio of 7, 2, and 1 respectively. To train the YOLOv3 model, we have tuned its parameters like subdivision, dimensions (width and height), max batches, and filters. Max batches and filters are updated according to formulae (7) and (8). Both parameters are highly dependent on the number of classes (*ncl*) in the dataset.


(7) Max Batches=ncl×2000

(8) Filters=(5+ncl)×3

The performance of the YOLOv3 version is evaluated with several configuration settings for different cases. In each case, filters and max batches are kept the same with values of 27 and 8000 respectively. Hyper-parameters used for tuning the model are shown in **Table 1**.

**Table 1:** Hyper-parameters used for training of YOLOv3.

Cases	Subdivisions	Width	Height	Max Batches	filters
Case 1	16	416	416	8000	27
Case 2	16	416	416	8000	27
Case 3	16	416	416	8000	27
Case 4	16	384	384	8000	27
Case 5	32	384	384	8000	27
Case 6	32	512	512	8000	27
Case 7	32	384	384	8000	27

**Figure 6:**
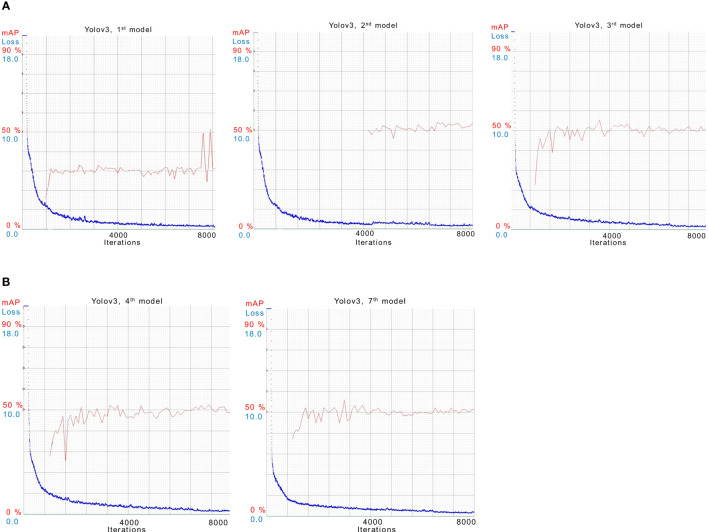
Illustration of training 1*^st^*, 2*^nd^*, 3*^rd^*,4*^th^* and 7*^th^* models for YOLOv3 in **(A)** and **(B)**.

In case 1*^st^* and 6*^th^*, the dataset used was not masked. Whereas, in case 2 *^nd^* and 5 *^th^*, LAB transformation is used. In case 3 *^rd^* and 7 *^th^* HSV transformation is used. It can be seen in the results that there is a slight variation in the mAP values but overall performance remains the same. In **Figure 6**, training results of all models other than the best models are presented.

**Figure 7:**
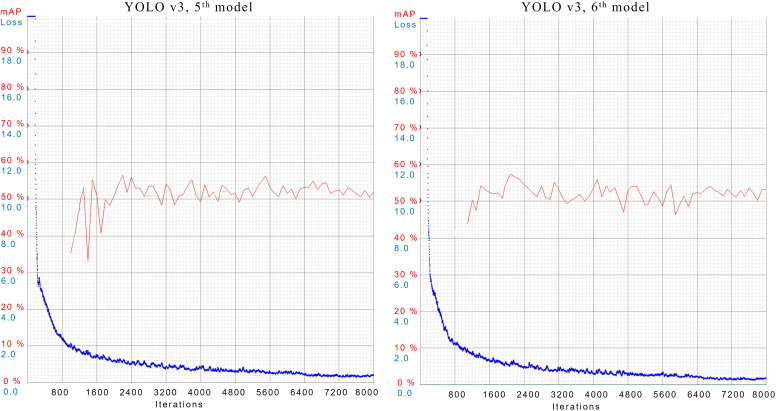
AL and mAP graphs for YOLOv3 models with 5*^th^* and 6*^th^* configuration setups.

In case 5*^th^* and 6*^th^*, both models have achieved the best score among all implementations. In both cases, Models are configured with the same subdivisions of 32 but different dimensions of 384 × 384 and 512 × 512 respectively. Their results during training are shown in **Figure 7**.

**Figure 8:**
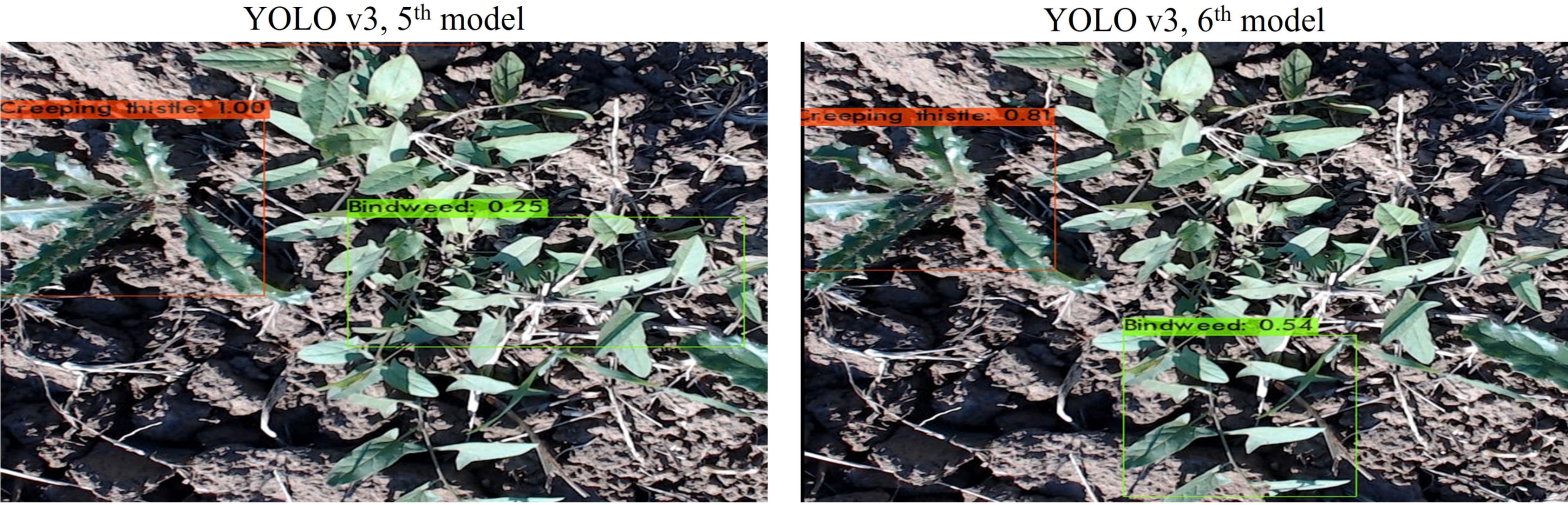
Inferencing results of 5*^th^* and 6*^th^* models of YOLOv3.

YOLOv3, 5*^th^* model has provided mAP of 52.0%, best mAP of 57%, and AL of 0.4076. Whereas 6*^th^* model has gained an mAP of 53.2%, the best mAP of 57%, and AL of 0.3394. Trained models are validated using the same test data. Inference results are shown in **Figure 8**, where YOLOv3’s 5*^th^* model has detected more objects than the 6*^th^* model.

For the evaluation of YOLOv3, we have prepared an unseen dataset, that dataset was not used for model training. In **Table 2**, the inference results obtained by evaluating the best model on the validation dataset are shown.

**Table 2:** Shows performance of best YOLOv3 model on test dataset.

Classes	Precision	Recall	F1-score
Grass	1.00	1.00	1.00
Creeping thistle	0.96	0.99	0.97
Bindweed	0.96	0.86	0.90
California poppy	0.97	1.00	0.99

Models trained in case 3*^rd^* and 4*^th^* have detected more objects of Grass and Creeping Thistle. In case 7*^th^*, the model trained has detected the highest number of objects of Bindweed and California poppy. On average, models trained in case 3*^rd^* and 4*^th^* have detected more objects of every class as compared to other cases. In **Table 3**, mAP and AL values for each case are shown.

**Table 3:** Shows AL, mAP, and Best AP of each model while training.

Cases	AL	mAP	Best AP
Case 1	0.1888	31.70%	52.00%
Case 2	0.3677	52.19%	54.00%
Case 3	0.4033	49.00%	52.00%
Case 4	0.3726	50.95%	55.00%
Case 5	0.4074	52.00%	57.00%
Case 6	0.3394	53.20%	57.00%
Case 7	0.4107	51.50%	56.00%

### 4.3 YOLOv3-tiny implementation

In the implementation of YOLOv3-tiny, we evaluated its performance in different configuration settings. In the configuration, parameters like maximum batches and filters, calculated by equation (7) and (8), are kept the same in each implementation as they depend on the number of classes in the dataset. In each case, filters and max batches are set to 27 and 8000 respectively. Change in other parameters is given in **Table 4**.

**Table 4:** Hyper-parameters used for training of YOLOv3-tiny.

Cases	Subdivisions	Width	Height	Max Batches	filters
Case 1	16	416	416	8000	27
Case 2	16	384	384	8000	27
Case 3	32	384	384	8000	27
Case 4	32	416	416	8000	27
Case 5	32	512	512	8000	27
Case 6	32	384	384	8000	27
Case 7	16	608	608	8000	27

In case 1*^st^* and 3*^rd^* the dataset used was not masked. Whereas, in case 2*^nd^* and 4*^th^*, LAB transformation is used. In case 5*^th^* and 7*^th^* HSV transformation is used. It can be seen in the results that there is a slight variation in the mAP values but overall performance remains the same.

**Figure 9:**
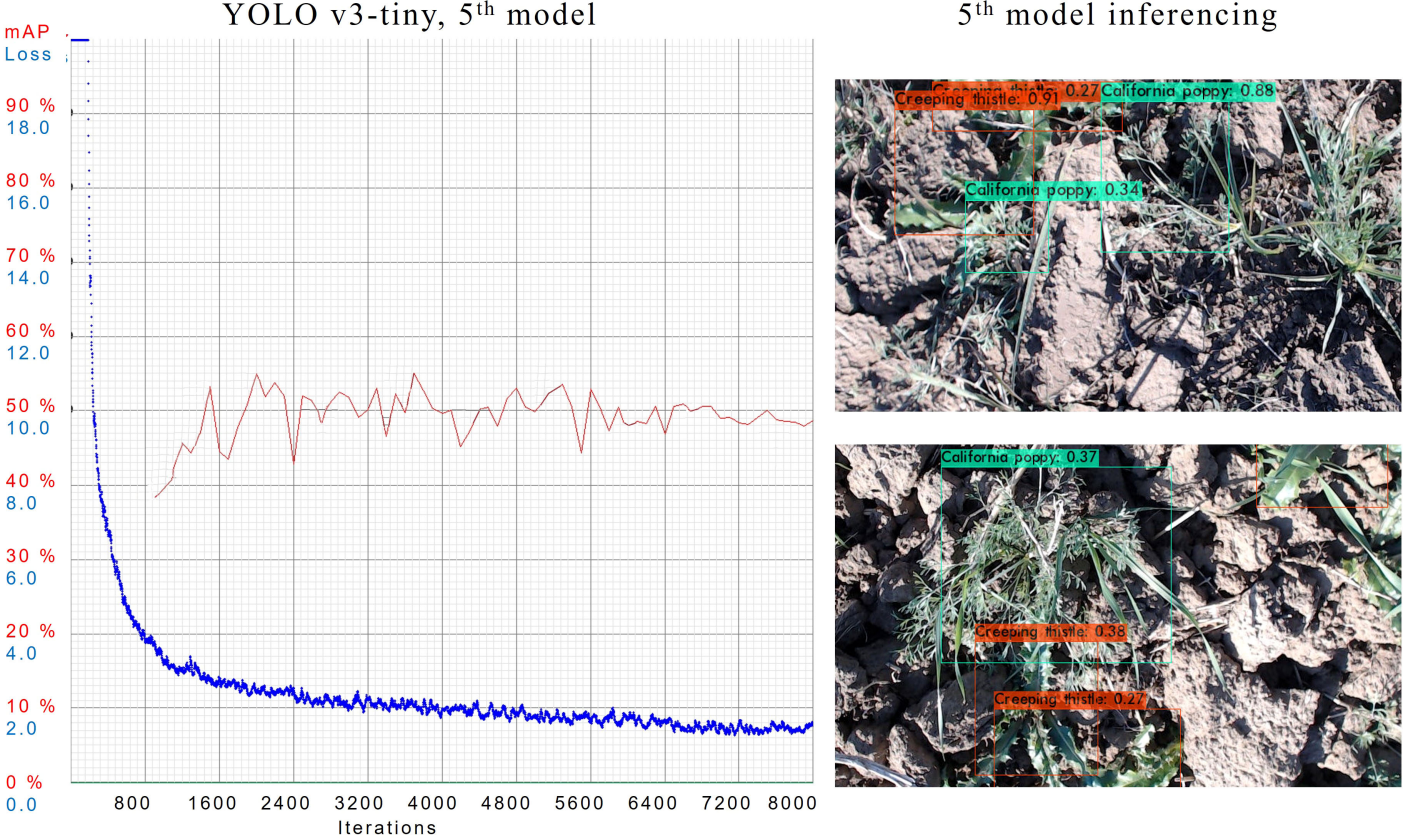
Training graphs and inferencing results of YOLOv3-tiny’s 5*^th^* model trained.

In 5*^th^* case, the model is configured with subdivisions of 32 and dimensions of 512 × 512. Training of this model is illustrated in **Figure 9**. The model has an mAP of 48.7%, the best mAP of 55% and an AL is 1.5265. In the above figure, we can see that 5*^th^* model is able to detect quite a number of objects with a high confidence score while evaluating the test dataset.

To evaluate YOLOv3-tiny in each case, we have validated models on an unseen dataset and calculated the correctly predicted objects. Inferencing results for the best case is shown in **Table 5**. Models trained in 1*^st^* and 2*^nd^* cases can detect more objects than the rest. Objects detected in 4*^th^*, 5*^th^* and 6*^th^* cases are the lowest in Grass, Bindweed, and California poppy.

**Table 5:** Shows performance of best model of YOLOv3-tiny on test dataset.

Classes	Precision	Recall	F1-score
Grass	0.98	1.00	0.99
Creeping thistle	0.94	0.99	0.97
Bindweed	0.97	0.74	0.83
California poppy	0.96	0.97	0.97

In **Table 6**, mAP and AL values for each case are shown.

**Table 6:** Shows AL, mAP and Best AP of each model trained in different cases.

Cases	AL	mAP	Best AP
Case 1	1.5944	31.70%	52.00%
Case 2	1.6950	52.19%	54.00%
Case 3	1.5103	49.00%	52.00%
Case 4	1.6289	50.95%	55.00%
Case 5	1.5265	52.00%	57.00%
Case 6	1.2477	53.20%	57.00%
Case 7	1.3618	51.50%	56.00%

### 4.4 YOLOv4 implementation

In the implementation of YOLOv4, we evaluated its performance in different configuration settings. We have tuned each model with parameters max batches, filters, subdivisions, and dimensions. Max batches and filters are calculated by using equations (7) and (8), whereas subdivisions and dimensions are different for each case as shown in **Table 7**.

**Table 7:** Hyper-parameters used for training of YOLOv4.

Cases	Subdivisions	Width	Height	Max Batches	filters
Case 1	16	384	384	8000	27
Case 2	16	384	384	8000	27
Case 3	16	608	608	8000	27
Case 4	32	416	416	8000	27

In 1*^st^* and 2*^nd^* case, models are configured with subdivision of 16, 384 × 384 width and height, max batches of 8000, and filters 27. Maximum batches and filters are the same for each case, as they depend on the number of classes the dataset has. In case 1*^st^*, the model has an mAP of 53.6%, best mAP of 60%, and AL of 2.1259. In case 2*^nd^*, the model has an mAP of 53.1%, best mAP of 63%, and AL of 2.3958.

In the case of 1*^st^* and 2*^nd^* models are trained on LAB and HSV datasets. We have analyzed that 1*^st^* model can detect more objects in the image than the 2*^nd^*. The mean average precision of both models is quite similar. **Table 8** shows the results of YOLOv4’s best model.

**Table 8:** Shows performance of best YOLOv4 model on test dataset.

Classes	Precision	Recall	F1-score
Grass	1.00	1.00	1.00
Creeping thistle	0.99	1.00	1.00
Bindweed	1.00	0.98	0.99
California poppy	0.96	1.00	0.98

In **Table 9**, mAP and AL values for each case are shown. We can see that although the best mAP values of case 1 and 2 are relatively high the mAP values of both cases is about the same. The AL of both cases is also the same. In the 3*^rd^* case, although we have got less mAP as compared to other cases the AL is also the lowest among these experiments.

**Table 9:** Shows AL, mAP, and Best AP of each model trained in different cases.

Cases	AL	mAP	Best AP
Case 1	2.1295	53.60%	60.00%
Case 2	2.3956	53.10%	63.00%
Case 3	1.7749	48.50%	57.00%
Case 4	1.85	73.10%	74.00%

**Figure 10:**
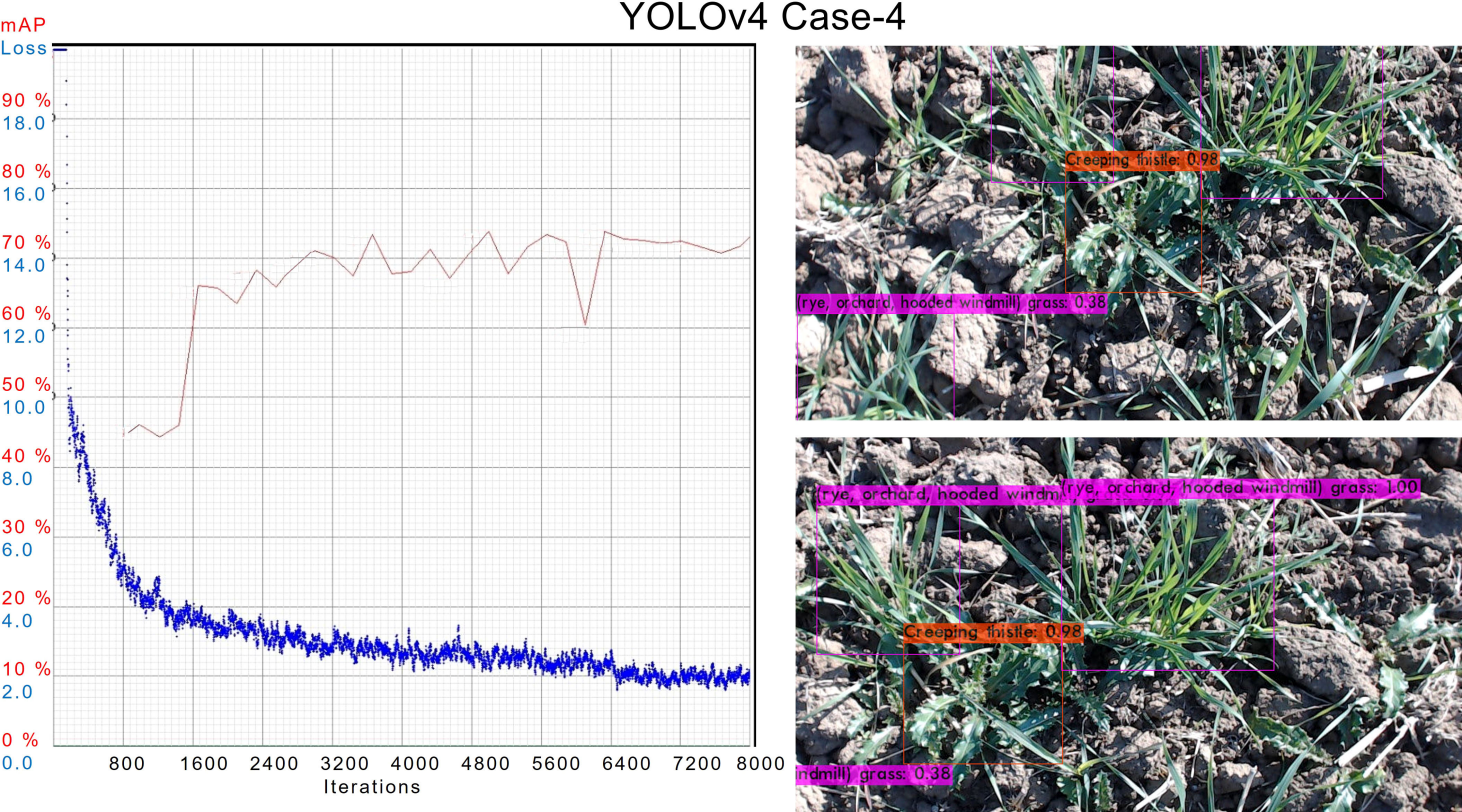
AL and mAP graph for the best YOLOv4 model with 4*^th^* configuration setups.

In case 4*^th^*, during training the performance of the trained model was best compared to all, as shown in **Figure 10**. The AL of this case is also among the lowest end. The average mAP remains above 70% which is the best so far. In the inference, the model has also outperformed others in the detection of objects.

### 4.5 YOLOv4-tiny implementation

YOLO model version four tiny is a small DL-based architecture. The rate at which it can detect objects is faster than the YOLOv4 version. But it has a drawback of detecting objects with lower accuracy. The model produced after its training is of very small size, which is mainly required for machines having lower computational power.

In the implementation of YOLOv4-tiny, we evaluated its performance in different configuration settings. In each setting, parameters like filters, max batches, dimensions, etc. are modified. Filters and max-batches are calculated by using equations (7) and (8) respectively, while values of other parameters are variable in every case as given in **Table 10**.

**Table 10:** Hyper-parameters used for training of YOLOv4-tiny.

Cases	Subdivisions	Width	Height	Max Batches	filters
Case 1	16	384	384	8000	27
Case 2	16	512	512	8000	27
Case 3	32	384	384	8000	27
Case 4	32	416	416	8000	27
Case 5	32	384	384	8000	27
Case 6	16	608	608	8000	27

**Figure 11:**
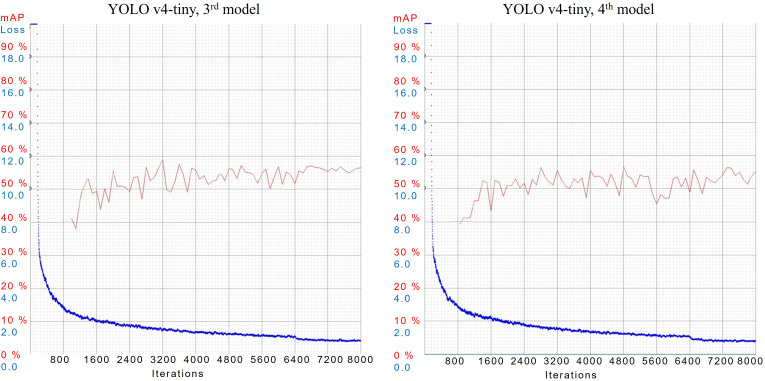
AL and mAP graphs for YOLOv4-tiny models with 3*^rd^* and 4*^th^* configuration setups.

During the training of YOLOv4-tiny in case 3*^rd^* and 4*^th^*, models are configured with 32 subdivisions and dimensions of 384 × 384 and 416 × 416 respectively. In case 3*^rd^*, the model has an mAP of 56.5%, best mAP of 59%, and AL of 0.8718. While in case 4*^th^*, the model has an mAP of 54.9%, best mAP of 57%, and AL of 0.8083. AL and mAP graphs of both cases are shown in **Figure 11**.

**Figure 12:**
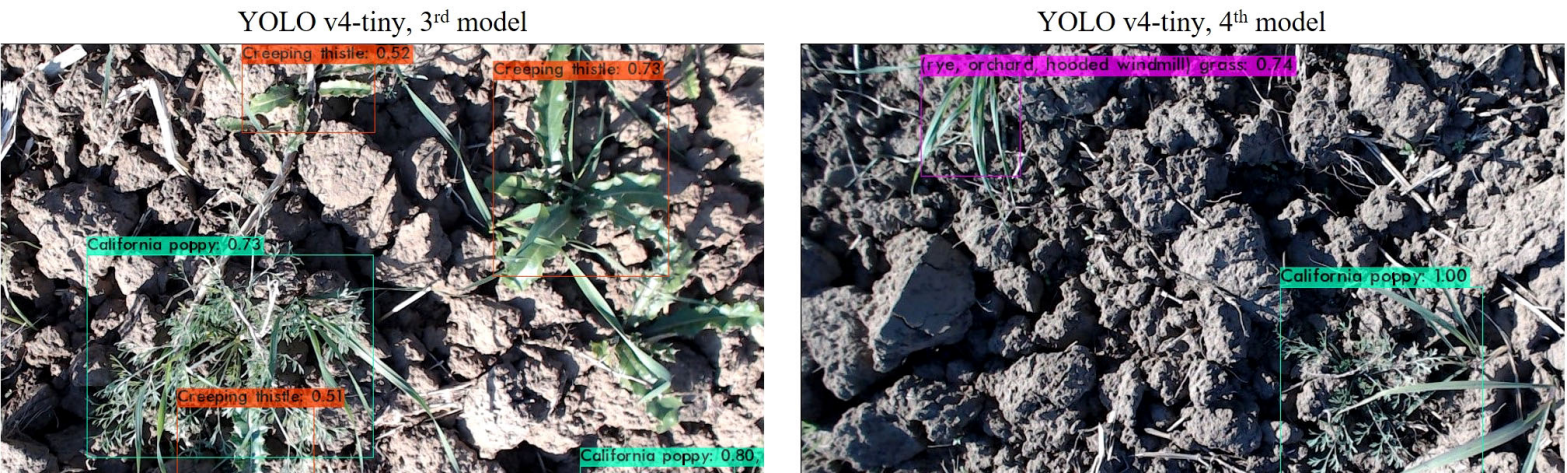
Inferencing results of 3*^rd^* and 4*^th^* models of YOLOv4-tiny.

Inferencing results of case 3*^rd^* and 4*^th^* are illustrated in **Figure 12**, where both models are quite efficient in predicting objects with a high confidence score. Both models have provided an average confidence score of 90% while evaluating them on unseen images.

Every model in YOLOv4-tiny’s implementation has performed outstandingly while predicting the object in the image. In **Table 11**, the results of the best model of YOLOv4-tiny are shown. The model’s performance in 5*^th^* case is the weakest as it has detected the least number of objects in the validation dataset. In **Table 12**, mAP and AL values for each case are shown.

**Table 11:** Shows performance of YOLOv4-tiny on test dataset.

Classes	Precision	Recall	F1-score
Grass	1.00	1.00	1.00
Creeping thistle	0.96	1.00	0.98
Bindweed	1.00	0.91	0.95
California poppy	0.95	0.99	0.97

**Table 12:** Shows AL, mAP and Best AP of each model trained in different cases.

Cases	AL	mAP	Best AP
Case 1	0.8089	54.00%	57.00%
Case 2	0.7774	55.00%	56.00%
Case 3	0.8718	57.00%	59.00%
Case 4	0.8083	55.00%	57.00%
Case 5	0.6552	54.00%	55.00%
Case 6	0.8441	53.00%	56.00%

## 5 Discussion

In this section, we have done an analysis of the performance of four YOLO variants (v3, v3-tiny, v4, and v4-tiny). Firstly, the results obtained in the training phase are analyzed. Secondly, the performance of each model while inferencing is discussed, and then we examined the impact of data augmentation and image processing on training and evaluation. Lastly, we have done a comparative analysis of the performance of our model with related work.

In the training phase, each YOLO variant is trained on the same system to have a neutral performance comparison. We have analyzed the difference in training performance by changing configuration parameters. In each case of implementation, models are configured with a combination of dimensions (384, 416, 512, 608) and sub-divisions (16, 32). It is observed that between all combinations there is a slight performance difference in mAP values, but a major difference in GPU’s memory usage. Decreasing subdivisions and increasing width and height can consume a lot of GPU memory. Among all YOLO variants, YOLOv4 has provided the best mAP and AL values. It has managed to identify all objects correctly, out of 102 total objects, with an average confidence score of 88.67%.

**Figure 13:**
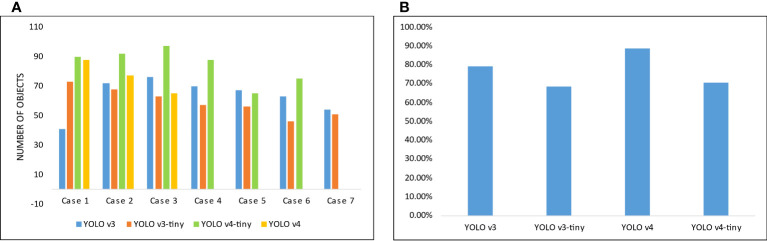
**(A)** Illustration of the number of objects detected by each model in different cases, **(B)** Mean confidence score of the best model of each YOLO variant.

In the evaluation of trained models, YOLOv4-tiny can identify more objects in overall every scenario. Behind it, YOLOv4 has achieved second best predictions rate, while YOLOv3 and YOLOv3-tiny have provided about the same results in most of the cases. In **Figure 13**, the number of objects detected by each model is given in (A) and the mean confidence score of the best models trained is illustrated in (B). It is observed that among all the implemented YOLO versions, YOLOv4 has provided us with the best confidence score while predicting.

**Figure 14:**
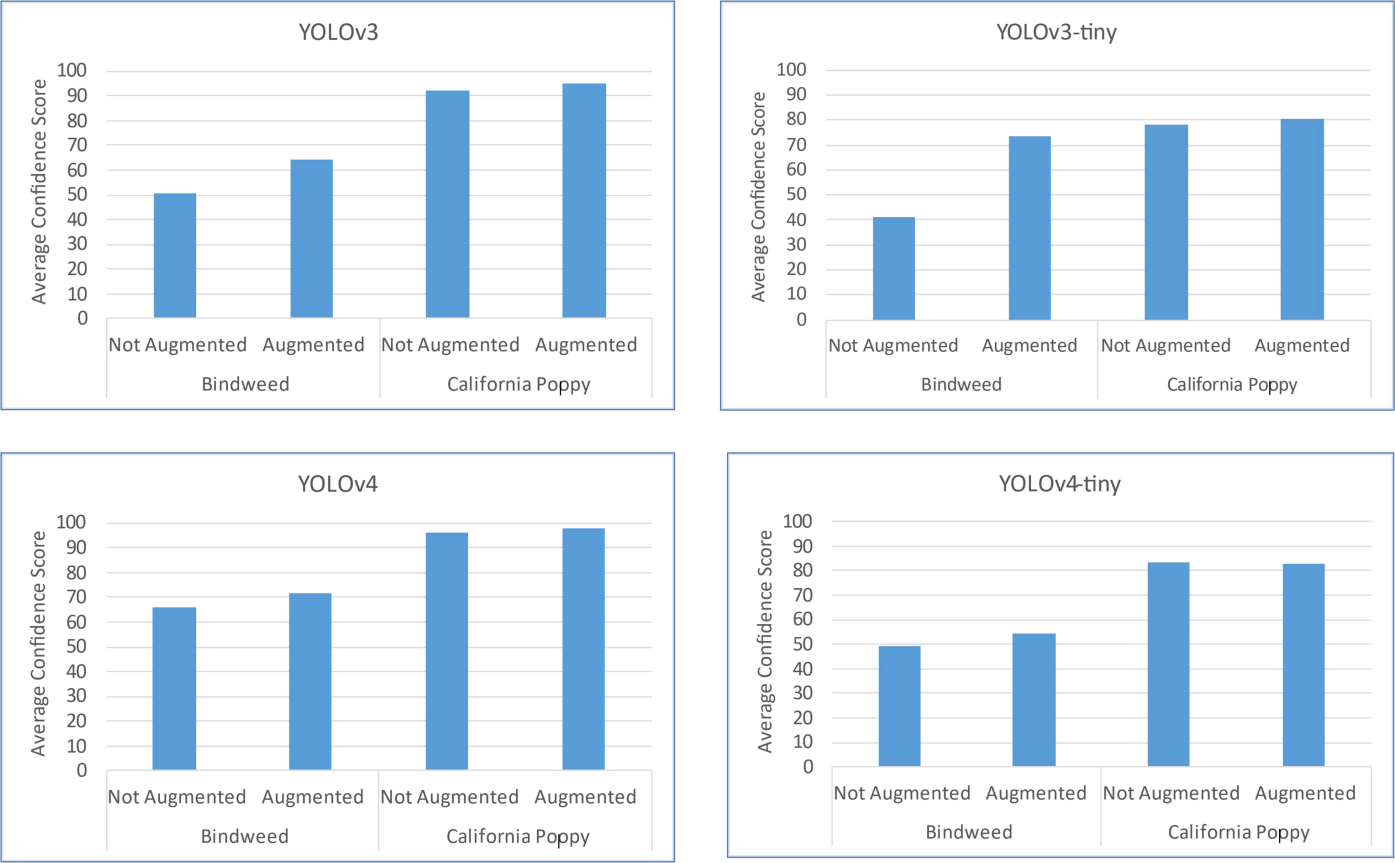
Change in each model’s results by the application of data augmentation.

It is evaluated that by data augmentation the confidence score and the number of objects correctly detected of two classes, Bindweed, and California poppy, has been increased. The augmentation outcome is illustrated in **Figure 14**.

In the above figure, an increase in confidence score can be seen after augmentation. A major increment for the Bindweed class is produced by YOLOv3-tiny. While the difference in California poppy’s results is very subtle. While application of LAB and HSV transformation to mask plants do not have a major impact on performance (in terms of mAP) and also it can potentially increase the processing cost, time, and resources. The YOLOv4 has detected objects with high confidence scores in the prediction process regardless of the data provided. So, we can deduce that YOLOv4 has the capability of detecting objects efficiently despite of insufficient quantity of data. We can see a comparative analysis of the performance of our model with the related work in the **Table 13**.

**Table 13:** Shows comparative analysis with related studies.

Ref	DL model	Precision	Recall	F1-score	mAP
(Ours)	YOLOv4	0.9875	0.9950	0.9925	73.10 %
(Partel et al. (2019))	YOLOv3	0.5900	0.4400	0.5000	59.00 %
(Madsen et al. (2020))	FRCNN	–	–	–	37.01 %
(Gao et al. (2020))	YOLOv3	0.9100	0.9800	0.9400	76.01 %
(Sivakumar et al. (2020))	FRCNN	0.6500	0.6800	0.6600	85.00 %
(Zhuang et al. (2022))	YOLOv3	–	–	0.9600	58.00 %
(Jin et al. (2022))	YOLOv3	0.9710	0.9700	0.9710	40.00 %
(Etienne et al. (2021))	YOLOv3	–	–	–	45.13 %
(Rajalakshmi et al. (2021))	YOLO (version NA)	–	–	–	83.00 %

## 6 Conclusion

The use of DL has greatly soared the performance of object detection systems. Detection of objects like weeds in the real field has been a challenging task because of the highly variable environment. In this study, we have proposed a DL-based weed detection model for the identification of weeds in real time. For this purpose, a real field dataset was collected and various data preprocessing techniques were applied to it, before using it as an input for training. Four YOLO versions (v3, v3-tiny, v4, v4-tiny) are implemented using different configuration settings to provide a comparative analysis of their inference results on unseen data.

We concluded that models configured with subdivisions of 16 and dimensions of 416 × 416 can generalize better on unseen data, predict more objects, and gives more accuracy and mAP as compared to other configurations. Data augmentation has also impacted greatly the performance. The model trained on augmented data has detected twice the number of objects as compared to other models. Meanwhile, the difference in performance upon implementation of LAB and HSV image transformation for masking plants is low.

We have analyzed that the best training results are provided by YOLOv4 architecture with an mAP of 73.1% and an average loss rate of 1.8. This model has achieved an accuracy of 98.88% by calculating the number of correctly predicted weeds in the unseen dataset. This model has the capability of being deployed in a real field to detect weeds.

In the future, we plan to build a variable rate spraying system for real-time weed management using the proposed model. In addition to this, we will add a variety of weed data and will also use other latest DL models to improve detection accuracy.

## Data availability statement

The datasets presented in this study can be found in online repositories. The names of the repository/repositories and accession number(s) can be found below: https://github.com/Aqib-Soft-Solutions/Wheat-Crop-Weeds-Dataset.

## Author contributions

Conceptualization, MA and MT; methodology, MA and MS; software, MS and MA; validation, MS and MA; investigation, MS, MA, and YH; resources, MA and MT; data curation, MS and MA; writing—original draft preparation, MS, MA; writing—review and editing, MA, MT, and YH; visualization, MS and MA; supervision, MA, YH, and MT. All authors contributed to the article and approved the submitted version.
